# *DatView*: a graphical user interface for visualizing and querying large data sets in serial femtosecond crystallography

**DOI:** 10.1107/S1600576719012044

**Published:** 2019-10-31

**Authors:** Natasha Stander, Petra Fromme, Nadia Zatsepin

**Affiliations:** a School of Molecular Sciences, Arizona State University, Tempe, AZ 85287, USA; b Biodesign Center for Applied Structural Discovery, Arizona State University, Tempe, AZ 85287, USA; c Department of Physics, Arizona State University, Tempe, AZ 85287, USA; d ARC Centre of Excellence in Advanced Molecular Imaging, Department of Chemistry and Physics, La Trobe Institute for Molecular Science, La Trobe University, Victoria 3086, Australia

**Keywords:** serial femtosecond crystallography, visualization, X-ray free-electron lasers, data analysis, graphical user interfaces

## Abstract

*DatView* is a new graphical user interface (GUI) for plotting parameters to explore correlations, identify outliers and export subsets of data. It was designed to simplify and expedite analysis of very large unmerged serial femtosecond crystallography (SFX) data sets composed of indexing results from hundreds of thousands of microcrystal diffraction patterns. However, *DatView* works with any tabulated data, offering its functionality to many applications outside serial crystallography. In *DatView*’s user-friendly GUI, selections are drawn onto plots and synchronized across all other plots, so correlations between multiple parameters in large multi-parameter data sets can be rapidly identified. It also includes an item viewer for displaying images in the current selection alongside the associated metadata. For serial crystallography data processed by *indexamajig* from *CrystFEL* [White, Kirian, Martin, Aquila, Nass, Barty & Chapman (2012 ▸). *J. Appl. Cryst.* **45**, 335–341], *DatView* generates a table of parameters and metadata from stream files and, optionally, the associated HDF5 files. By combining the functionality of several commonly needed tools for SFX in a single GUI that operates on tabulated data, the time needed to load and calculate statistics from large data sets is reduced. This paper describes how *DatView* facilitates (i) efficient feedback during data collection by examining trends in time, sample position or any parameter, (ii) determination of optimal indexing and integration parameters via the comparison mode, (iii) identification of systematic errors in unmerged SFX data sets, and (iv) sorting and highly flexible data filtering (plot selections, Boolean filters and more), including direct export of subset *CrystFEL* stream files for further processing.

## Abstract 2

*DatView* is a new graphical user interface for simplifying analysis by facilitating plotting parameters and exporting selections from large serial crystallography data sets.

## 1. Introduction   

Serial femtosecond crystallography (SFX) at X-ray free-electron lasers (XFELs) is a recent innovation in macromolecular crystallography where structures are determined from thousands of snapshot diffraction patterns from nano/microcrystals delivered to a pulsed XFEL beam in a serial fashion. The short (femtosecond) pulse duration diminishes the effect of radiation damage on the data because each crystal is exposed only once in a diffraction-before-destruction approach (Neutze *et al.*, 2000 ▸; Chapman *et al.*, 2011 ▸; Spence, 2017 ▸). To account for shot-to-shot variation in both crystals and the XFEL beam, SFX data sets are often large, with up to hundreds of thousands of diffraction patterns. These large data sets are typically reprocessed many times with software such as *CrystFEL* (White *et al.*, 2012 ▸) or *cctbx.xfel* (Hattne *et al.*, 2014 ▸) to find optimum indexing, integration and merging parameters. Data visualization is an important guide for this optimization, as well as for optimal data collection. While tools such as *cell_explorer* (White, Mariani *et al.*, 2016 ▸), the *Data Exploration Toolkit* (Zeldin *et al.*, 2015 ▸) and *cxiview* (Barty *et al.*, 2014 ▸) exist to give feedback during data collection and processing, up to now there has been no unified graphical user interface (GUI) for interactive visualization of SFX data sets.

*DatView*’s unified GUI increases the power of the program to explore correlations between many parameters simultaneously, export selections and compare the effects of different processing approaches. It loads any text table where each column is a parameter and each row an item. Through the GUI, users select parameters to plot and then make selections directly on the plots. Selections on one plot are synchronized to all other plots, showing the current selection as opaque and the full data set as semi-transparent. An item viewer displays the items in the current selection and supports selections on an item-by-item basis. The available plots and options are described in detail in Section 3.

An important extension to data visualization is exporting the selection for further processing. This enables outlier rejection [motivated by Diederichs & Karplus (2013 ▸) and Assmann *et al.* (2016 ▸)], obtaining a consistent subset for advanced processing (Kabsch, 2014 ▸; White, 2014 ▸; Ginn *et al.*, 2015 ▸; Uervirojnangkoorn *et al.*, 2015 ▸) or splitting the data set into user-defined bins (Foadi *et al.*, 2013 ▸; Barends *et al.*, 2015 ▸; Zeldin *et al.*, 2015 ▸). *DatView* has three available export formats for selections: (i) a text table, the *DatView* input format (.dat), (ii) a file list (.lst) and (iii) *CrystFEL* unmerged indexing output, called a stream file, which contains each pattern’s partially integrated reflections, unit cell, orientation, diffraction resolution and peaks used for indexing, with the associated indexing and integration parameters, and other metadata (.stream) (White *et al.*, 2012 ▸). Export options are described in Section 4.

*DatView* has a comparison mode to support frame-by-frame parameter optimization, similarly to *IOTA* (Lyubimov *et al.*, 2016 ▸). For the comparison mode, multiple input tables (indexing results from different indexing conditions, for example) are combined with information about which diffraction patterns are equivalent across the different input files. From the GUI, users can plot the value of a parameter from one input table against the same parameter from the equivalent pattern of a different input table. Users can then export a data set optimized with respect to a chosen output parameter (*e.g.* diffraction resolution) by using the minimum or maximum filter available in *DatView*’s comparison mode. A new stream file is written, containing the optimal indexing result for each crystal, extracted from the multiple stream files from the multiple indexing attempts. The comparison mode is described in Section 5.

*DatView* is a standalone program. It is written in Python 3 and available from https://github.com/nstander/DatView, with documentation at https://zatsepinlab.atlassian.net/wiki/spaces/DAT/overview. It uses the PyQt, matplotlib, NumPy, SciPy, h5py, pyqtgraph and lxml libraries. It additionally uses a modified geometry file parser from the CfelPyUtils library and the histogram clip functionality of *cxiview*, which are both included with the code.

## 2. Input   

*DatView* loads text tables, so it can be used for many types of data set, although a preprocessing step may be required. For *CrystFEL* stream files or list files, the script datgen.py writes a table with parameters from the input file(s) and, optionally, parameters from HDF5/CXI image files or NumPy arrays. This table is the input for *DatView*. The optional parameters make it easy to include information like time stamps, XFEL pulse durations and sample information in the table. The table is an index into both the stream file and the image file, allowing the item viewer to display the images from the image file and overlay found and predicted peaks from the stream file. Table files can be combined, so preprocessing can be run in parallel for individual input stream files, and streams do not need to be duplicated in a single large stream file for display and calculation of basic statistics. The table index is generally smaller than the input stream files, which reduces the loading time of *DatView* compared with programs like *cxiview* (Barty *et al.*, 2014 ▸) or *cell_explorer* (White, Mariani *et al.*, 2016 ▸) (see Table 1 ▸ for a comparison). However, the update time in the GUI will depend on the size of the input file and the number of plots. *DatView* is slower than *cell_explorer* because *DatView* is written in Python 3 rather than C (as is the case for *cell_explorer*) and is typically managing more parameters.

**Figure 1:**
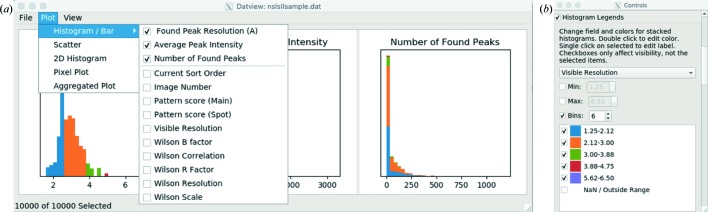
(*a*) The main window of *DatView*, containing histograms, three per row, with additional rows appearing as needed. Additional plots appear in their own windows. (*b*) The control panel interface for stacked histograms. This is a hit-finding data set from NSLS-II (unpublished data).

Preprocessing scripts have also been developed for Pandas HDF5 spectroscopy files at PAL-XFEL (Ko *et al.*, 2017 ▸) [used in Fig. 4(*b*)] and for serial crystallography hit finding on the Frontier Microfocusing Macromolecular Crystallography (FMX) beamline at NSLS-II (Fuchs *et al.*, 2014 ▸), where data are collected on an EIGER 16M detector (Könnecke *et al.*, 2015 ▸; Casanas *et al.*, 2016 ▸). Data from NSLS-II are used in Figs. 1 ▸ and 6. In addition to formatting the data as a text table, the external configuration file may need to be edited. The external configuration file describes the data type of each parameter so it can be loaded by NumPy, and is necessary for any data containing non-numeric fields. It also allows the configuration of many other parameters such as color maps, default histograms and displays in the item viewer. The preprocessing script and configuration file for Pandas HDF5 spectroscopy files at PAL-XFEL are shipped with *DatView*, and NSLS-II files are linked to from *DatView*’s GitHub page.

## 3. Data visualization   

*DatView* provides synchronized interactive plots and an item viewer. The main window contains histograms, three per row, with additional rows appearing as needed [*e.g.* Fig. 1 ▸(*a*)]. The default histograms to open are given in the external configuration file. For SFX, the unit-cell parameters are opened by default. Additional plots are available from the plot menu and detailed below. The suggested parameters for those plots are also configurable, and for SFX are set to relevant parameters such as the predicted detector shifts and the unit-cell volume against sorted order. Options such as the stacking and colors of histograms can be configured in the control panel available from the View menu [Fig. 1 ▸(*b*)]. The item viewer (Section 3.3 and Fig. 4) is also available from the View menu. Exporting (Section 4) is part of the File menu.

### 3.1. Synchronization   

**Figure 2:**
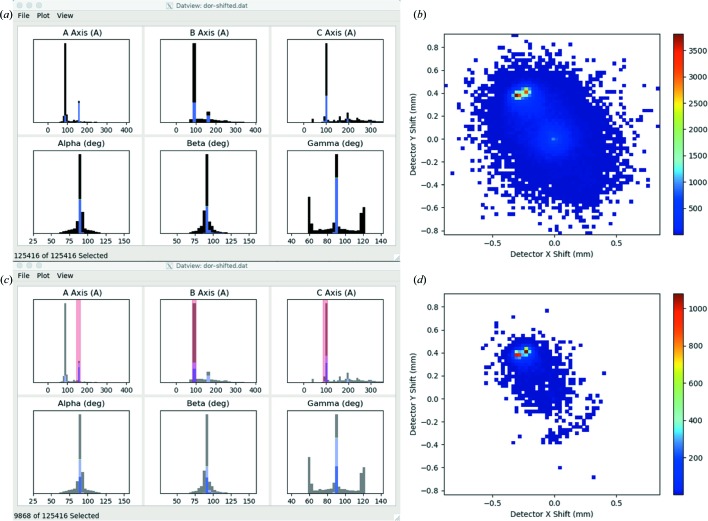
Data from CXIDB ID 40 (Fenalti *et al.*, 2015 ▸; White, Barty *et al.*, 2016 ▸) indexed with an incorrect beam center. (*a*) The initial main window of *DatView*, showing histograms of the unit-cell distributions colored by centering (blue is *C* centered, black is *P* centered). (*b*) A 2D histogram of the predicted detector shifts from indexing, generated from the Plot menu. (*c*) Selecting regions of the unit-cell distribution with Shift + click and drag updates panel (*d*) to emphasize the detector shift needed for the correctly indexed patterns.

*DatView* synchronizes the current selection to all plots, making it easy to simultaneously visualize correlations across any number of parameters on multiple plots in one, two and three dimensions. For SFX, this can facilitate solving problems like the determination of the correct beam center. As an example, SFX data from the Coherent X-ray Imaging Data Bank (CXIDB) (Maia, 2012 ▸), data set ID 40 (Fenalti *et al.*, 2015 ▸; White, Barty *et al.*, 2016 ▸), were re-indexed with *CrystFEL* Version 0.7.0 with an incorrect beam center and no unit-cell constraints. This represents the start of an experiment where the exact geometry is often not known, but can be roughly estimated from indexing (typically some well known calibration sample) (White, 2019 ▸). *DatView* opens the table generated by datgen.py with histograms of the six unit-cell parameters stacked by centering (*C* centered is blue, *P* centered is black), as shown in Fig. 2 ▸(*a*), similarly to *CrystFEL*’s *cell_explorer* (White, Mariani *et al.*, 2016 ▸). Multiple peaks or very noisy unit-cell distributions are commonly a sign that the geometry is inaccurate.

A 2D histogram (where the coloring is by density) can be generated from the Plot menu [Fig. 2 ▸(*b*)], with the predicted detector shift in *x* and *y* selected as the horizontal and vertical axes for the plot, respectively, akin to that produced by the *CrystFEL* script *detector-shift*. The distribution of predicted detector shifts in this example is noisy, with multiple peaks. Here, *DatView*’s synchronized plots are useful because users can select regions from the unit-cell distributions with Shift + click and drag, and the detector-shift plot updates accordingly, thus quickly identifying what detector center correction needs to be applied to increase the indexing yield with the correct unit cell. Selecting regions corresponding to the expected unit cell [Fig. 2 ▸(*c*)] gives Fig. 2 ▸(*d*), emphasizing the peak near (−0.3, 0.4) which was the offset used in this example.

### 3.2. Interactivity   

**Figure 3:**
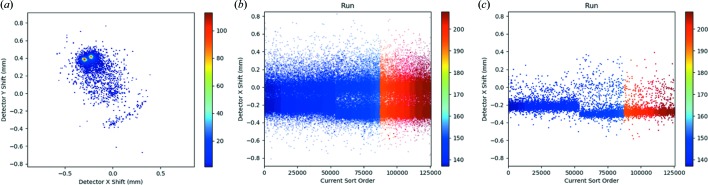
(*a*) The same 2D histogram of predicted detector shifts as in Fig. 2 ▸(*d*) but with finer binning, making the two distinct clusters easier to view. (*b*) A scatter plot of the detector *x* shift over sorted order (order of data collection) colored by run number, with the default display setting of showing the full data set semi-transparent and the current selection opaque. (*c*) The same as panel (*b*) but showing only the current selection, highlighting the point where the two clusters in panel (*a*) separate.

The interactivity of the plots also aids in data visualization. Histogram and 2D histogram binning can be changed from the context menu or with the keyboard shortcuts ‘+’ and ‘−’. Continuing the current example of determining the correct beam center, increasing the binning on the example detector-shift plot shown in Fig. 2 ▸(*d*) clarifies that there are two distinct clusters, as shown in Fig. 3 ▸(*a*). At this point, the user could select directly from the predicted detector-shift plot (shift + click and drag) to limit the selection to just one of the clusters and look for correlations in other variables. However, given that a change in beam center is often the result of a change in the experiment at a particular point in time, another logical step would be to plot the detector shift over time.

Scatter plots can be created from the plot menu with any parameters for the *x* and *y* axes and the color. To plot the detector shift over time, the predicted detector shift in *x* (since it appears larger than the shift in *y*) is used for the *y* axis, the current sort order is used for the *x* axis and the run number is used for color. The current sort order is configurable from the control panel, but will initially be the same as the file order. To sort in order of data collection (time), the image file name was used because the date and time are part of the file names for the example data. In the initial plot shown in Fig. 3 ▸(*b*), it is hard to see a clear shift because with over 100 000 patterns in the semi-transparent full data set the 9868 patterns correctly indexed in the opaque data set are not distinguishable. All plots in *DatView* can be changed to show only the current selection or the full data set (ignoring the current selection). Plotting just the current selection simplifies the plot [Fig. 3 ▸(*c*)], revealing an obvious change in the *x* shift.

All plots in *DatView* allow zooming with the scroll wheel and panning with click and drag. Additional information about data under the cursor is also available from the tooltips. By manipulating the example plot in Fig. 3 ▸(*c*), it can be determined that the shift occurs around pattern 53 500, which is approximately the break between runs 144 and 146. This may be correlated with changes in the experimental setup, such as the start of a new shift, changes in the XFEL beam that made realignment necessary, or a different sample-to-detector distance where the detector rails are slightly misaligned from the optical axis, as has been observed on a number of beamlines. Users may decide from this information to use a different beam center for the first part of the data set than for the second part.

### 3.3. Item viewer   

**Figure 4:**
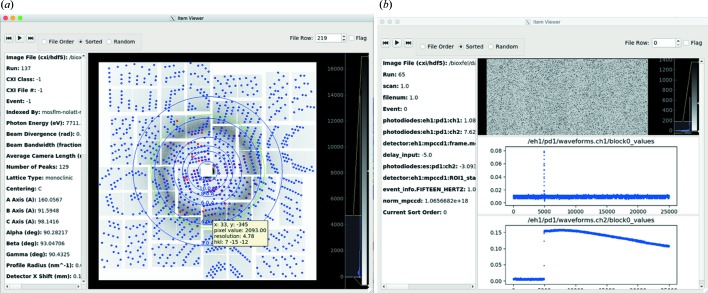
*DatView’s* item viewer. The left-hand pane shows all the statistics available for that row from the input table and the right-hand pane shows data from the HDF5 file. (*a*) A view of a pattern from CXIDB ID 40 (Fenalti *et al.*, 2015 ▸; White, Barty *et al.*, 2016 ▸). The image file information from the input table is used to display the image and the stream file information from the input table to overlay predicted (blue) and found (red) peak locations. A *CrystFEL* geometry file is used to lay out the information and for the resolution rings (blue) and the pattern’s diffraction resolution limit ring (green). (*b*) A frame of spectroscopy data collected at PAL-XFEL, showing readouts from an area detector and two photodiodes.

Selections are also synchronized to an item viewer. The item viewer (Fig. 4 ▸) has two panels. On the left is a list of all statistics and metadata in the input table for a particular row. On the right is a frame viewer that can display 1D or 2D data from HDF5/CXI files as scatter plots or images, respectively [see Fig. 4 ▸(*b*) for readouts from an area detector and two photodiodes from a PAL-XFEL spectroscopy data set]. For diffraction data [Fig. 4 ▸(*a*)], the viewer is similar to *cxiview* (Barty *et al.*, 2014 ▸) and can overlay found and predicted peaks from the stream file. The tooltip hover text displays the *x* and *y* coordinates and pixel value, as well as the resolution and Miller index, if known. Previous, Play and Next buttons provide a simple interface for viewing images in the current selection, and the flag check box allows changing the current selection. As an example, sometimes ice rings make the apparent diffraction resolution limit of a pattern abnormally high. The user could select the highest diffracting patterns from a histogram of the apparent diffraction resolution limit in the main window and then play through them in the item viewer, flagging frames that contain ice rings. Then, from the control panel, the user can set the selection to exclude any flagged items (or only include flagged items) to update the plots with statistics only from the clean data set.

### 3.4. Other plots   

**Figure 5:**
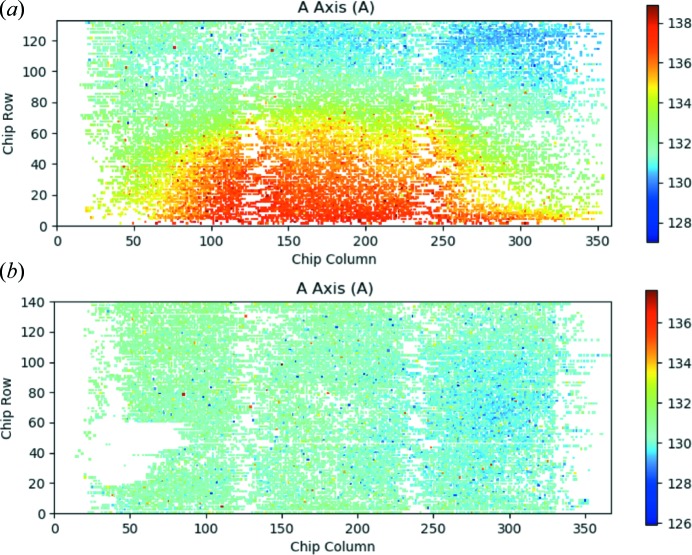
Example of *DatView* pixel plots, showing the *a* axis of the unit cells of membrane protein microcrystals by position on micropatterned silicon chips mounted on the high-speed Roadrunner goniometer (Roedig *et al.*, 2017 ▸) (unpublished data). Data collection began at the bottom of the chip. (*a*) A pixel plot showing the variation in unit-cell *a* axis that may be related to humidity or temperature changes during data collection, and (*b*) the *a*-axis pixel plot from another chip from the same experiment that did not show much variation in the unit cell.

Of the five plot types currently available in *DatView*, three have already been introduced in the previous examples: histograms, 2D histograms and scatter plots. The remaining two plot types are pixel plots and aggregated plots. Pixel plots were developed for visualizing data from microcrystals on a fixed target that is raster scanned across the beam. Two examples are shown in Fig. 5 ▸ from data collected from membrane protein microcrystals on micropatterned silicon chips mounted on the high-speed Roadrunner goniometer (Roedig *et al.*, 2017 ▸) (unpublished data). The color of the pixel at position (*x*, *y*) is determined by a user-selected parameter, which was the unit-cell *a* axis in this example. Data collection began at the bottom of the chip, so the large change in the *a* axis in Fig. 5 ▸(*a*) compared with Fig. 5 ▸(*b*) could indicate temperature or humidity changes during data collection. Such feedback during data collection can help optimize the experimental setup and make the best use of beamtime.

Aggregated plots bin one of the axes and display a calculated statistic of the bin on the second axis. For example, users can bin the data by run number and plot the average unit-cell volume of each bin, with optional standard-deviation error bars. A second dimension of binning can be added by splitting the data into multiple lines. An example is described in Section 5.1 and shown in Fig. 7(*a*).

## 4. Export   

While data visualization on its own is very useful to guide data-collection strategies and data processing, the data-export options in *DatView* are anticipated to be most useful for many users because they enable users to save the current selection for further processing, applying sorting and binning in a flexible and user-friendly way.

### 4.1. Export formats   

There are three available export formats: table, file list and *CrystFEL* stream file. The first is the input table format but outputting only the rows from the current selection. The second is a file list of image file names with one file name per line. For multiple-event HDF5/CXI image files, the event numbers are output following *CrystFEL* convention with ‘//’ separating the file name and event number on each line. This format can be used as input to *CrystFEL* auto-indexing. The final format is a *CrystFEL* stream file that can be used with *CrystFEL* merging programs (White *et al.*, 2012 ▸; White, Mariani *et al.*, 2016 ▸). The stream file output is only available with tables generated from stream files because it uses the index into the stream file to copy the text for each selected crystal into a new file. The stream file output will not match the *CrystFEL* convention for stream file(s) with multiple crystals for a frame because *DatView* repeats all frame information for each crystal. This convention is necessary to be able to select individual crystals from a multi-crystal frame and for sorting.

### 4.2. Export options   

**Figure 6:**
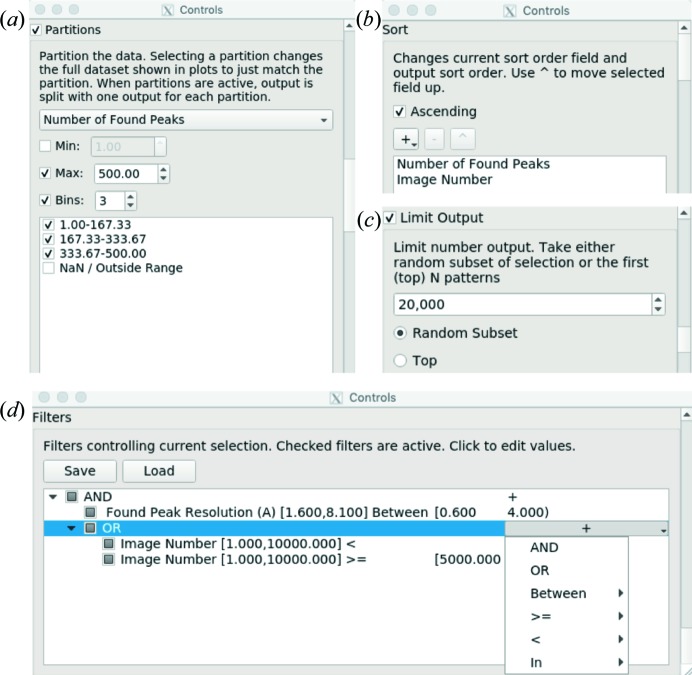
The control panel interfaces for (*a*) partitions controlling output bins, (*b*) sorting, (*c*) limiting output, and (*d*) creating and editing filters. The filter tree in panel (*d*) is equivalent to the filter in the text: (high resolution AND (pattern number < 2000 OR pattern number 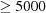)), showing how nested parentheses become nested layers in the tree structure. These windows use headings from NSLS-II hit-finding tables (unpublished data).

There are three main export options: sort, limit and partition [Figs. 6 ▸(*a*)–6 ▸(*c*)]. The exported selection follows the current sort order [Fig. 6 ▸(*b*)]. The sorted output enables users to try different cutoffs in *CrystFEL* merging programs using start-after and stop-after options, instead of duplicating information into many potential input files. There is also a limit option [Fig. 6 ▸(*c*)] so that users can export just the first *N* patterns of the current sort order. Alternatively, the limit can be used to select a random subset of size *N* from the current selection. This is useful if the user wishes to compare the results of different selections with equivalent numbers of patterns. Finally, partitions [Fig. 6 ▸(*a*)] enable users to bin data by any parameter and create a different output file for each bin. For instance, a European XFEL data set could be partitioned by pulse ID in the train of pulses to check for systematic changes (Wiedorn *et al.*, 2018 ▸) or to separate pulses before and after laser illumination for time-resolved pump–probe SFX.

### 4.3. Advanced selections   

*DatView* provides an interface for specifying exact cutoffs of selections for improved precision compared with drawing the cutoffs on a plot. These controls are available through the filter section of the control panel shown in Fig. 6 ▸(*d*). Plots are automatically connected to filters, so selecting a range on a plot is equivalent to typing that range into the filter interface. Any selection drawn onto a plot is represented with a ‘between’ filter because it enforces values on that parameter that are between the given minimum and maximum.

The filter interface includes additional controls for more complex selections. As an example, the user may want to select high-resolution patterns from the beginning and end of the data, avoiding a problematic section in the middle. As a combination of Boolean and relational operators, the desired result may look like this: (high resolution AND (pattern number < 2000 OR pattern number 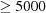)). Filters in *DatView* are represented as a tree, with each layer of parentheses represented by a nested layer in the tree. Nodes of the tree with children are the AND or OR operators that determine how the child nodes are combined. As an example, the condition above is shown in Fig. 6 ▸(*d*).

Leaf-node filters operate on the data. Three of these filters have already been mentioned: ‘between’, ‘less than’ and ‘greater than or equal to.’ A fourth available operator is 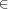 (in the set of), which is available for categorical variables. It displays possible values (categories) and the user can toggle each value’s inclusion individually. As an example, in the CXIDB ID 40 data set indexed with the incorrect beam center described above, there were two possible cell centerings (*C* and *P*), as seen in Fig. 2 ▸(*a*). There is a third possibility of no cell centering for patterns that were not indexed. An ‘in’ filter displays a drop down with these three possible centerings and the user can toggle each possible value individually. Additional filters are unique to the comparison mode and described in Section 5.2.

## 5. Comparison mode   

For advanced users and developers, *DatView* has a unique feature called comparison mode for visualizing the changes in parameters from different data-processing approaches. This requires a preprocessing script, datcompare.py, that takes multiple input tables and combines them into a .npz (compressed NumPy) file that is loaded directly with the viewer. This file includes every row from every input file and additional information linking equivalent frames from the different input files. As an example, the data set from CXIDB ID 40 was indexed with *CrystFEL* Version 0.7.0 with the provided scripts and input files. Then, it was indexed three additional times with different integration radii. The integration radii parameter in *CrystFEL*’s *indexamajig* defines the outer radius of a peak and the inner and outer radii of the peak’s local background. Comparison mode can be used to visualize how different integration radii impact the indexing results on a frame-by-frame basis.

### 5.1. Plots   

**Figure 7:**
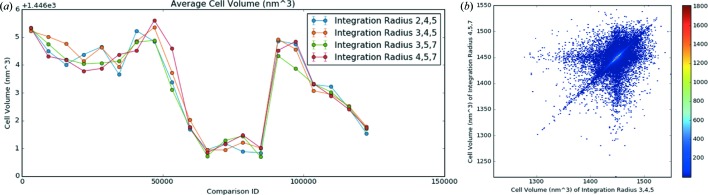
*DatView* comparison plots of indexing results from four different indexing conditions for SFX data from CXIDB ID 40 (Fenalti *et al.*, 2015 ▸; White, Barty *et al.*, 2016 ▸). The entire data set was processed with the original (published) integration radii of 3, 4, 5 pixels, then 2, 4, 5 pixels, 3, 5, 7 pixels and the *CrystFEL* default integration radii of 4, 5, 7 pixels. (*a*) An aggregated plot across equivalent frames showing the average cell volume grouped by processing. (*b*) A 2D histogram showing the apparent change in unit-cell volume when indexing with the integration radii (3, 4, 5 pixels) from the scripts downloaded from CXIDB and the default integration radii (4, 5, 7 pixels).

Two additional parameters for plots appear in comparison mode: the comparison ID, which is unique for each diffraction pattern irrespective of the stream files, and the comparison group, which identifies the input table of the frame. In the example, the comparison ID field range is from 0 to 125 415 so there is a unique value for each frame in the data set. The comparison group has four possible values for the four different input tables. To examine whether there is a change in the unit-cell volume determined by indexing with different integration radii, an aggregated plot can be used with the comparison ID for the *x* axis and a different line for each comparison group, shown in Fig. 7 ▸(*a*). From the plot, it is apparent that the integration radii influence the unit-cell volume since the different lines do not overlap, but the effect is not consistent for all patterns since the lines cross.

For a more detailed view of the changes, two additional plots are available, called comparison scatter and comparison 2D histogram. They are variants of the scatter and histogram plots where the *x* and *y* axes are input tables. Each point is a frame, with the *x* value corresponding to the value of the parameter in the *x* input table and the *y* value corresponding to the value of the parameter from the *y* input table. A comparison 2D histogram for the integration radii used in the initial scripts as the *x* axis and the default *CrystFEL* integration radii as the *y* axis is shown in Fig. 7 ▸(*b*).

### 5.2. Filters   

After visualizing the frame-by-frame changes from different processing approaches, it is useful to create a combined data set that is optimal for each frame. The software *IOTA* (Lyubimov *et al.*, 2016 ▸) does this by screening peak-finding parameters for each frame and using the parameters for each frame that minimize an optimization function. *DatView* enables this kind of processing with the ‘min’ and ‘max’ filter operators available in the comparison mode. These filters keep the frame from each set of equivalent frames that minimizes or maximizes the selected parameter. In the example, a minimum filter on the cell volume would result in a selection with 125 416 frames (if all frames were indexed in at least one input file), where each selected frame comes from the input table where its cell volume is minimal.

The ‘min’ and ‘max’ filters are comparison filters because they operate on each set of equivalent frames. In other words, the inclusion of a frame depends on the values of the parameter for that frame in the other input tables. There are four other comparison filters: ‘all between’, ‘any between’, ‘all same’ and ‘any different’. The ‘all between’ filter can be used to select the set of patterns that were indexed with all four integration radii by enforcing equivalent frames to have a unit-cell volume in the normal range. In the example, this results in 232 308 selected rows, meaning 58 077 patterns were indexed in all four conditions. The ‘any between’ filter on cell volume would keep any frame that was indexed under at least one condition. For the example data, this would result in 287 916 selected rows, meaning 71 979 patterns were indexed under at least one condition.

The ‘all same’ and ‘any different’ filters help with parameters that only change occasionally. For the example data set, the diffraction resolution limit calculated by *CrystFEL*’s *indexamajig* is not always affected by the integration radii. Using the ‘all same’ filter on the diffraction resolution limit for the set of patterns indexed under every condition reduces the selection from 232 308 to 138 384, showing that for 34 596 patterns the different integration radii did not have an impact on the calculated diffraction resolution limit. The ‘any different’ filter is the opposite of the ‘all same’ filter and results in the selection of the other 93 924 rows corresponding to the 23 481 patterns whose diffraction resolution limit was different in at least one input file.

## 6. Conclusions   

*DatView*’s GUI for visualizing data, exporting subsets and comparing processing methods makes data analysis for SFX data sets easier. The synchronized and interactive plots aid in diagnosing and fixing problems, and the item viewer makes it easy to make selections on a frame-by-frame basis. Export functionality enables users to create clean data sets or efficiently bin data sets into different files. The comparison mode allows basic frame-by-frame optimization of indexing and integration parameters. *DatView* is included in the CFEL software stack at the European XFEL, and has also been used on the FMX beamline at NSLS-II, at PAL-XFEL and at LCLS. It is anticipated that it will help many users make the most of their serial crystallography data and other types of multi-parameter data.

## Figures and Tables

**Table 1:** Data sizes and comparison of time taken (minutes:seconds) for the appearance of the GUI for *CrystFEL* stream files from data set CXIDB ID 40 (Fenalti *et al.*, 2015 ▸; White, Barty *et al.*, 2016 ▸) Data were processed with *CrystFEL* Version 0.7.0 and times were averaged over five repetitions. The small stream file is the entire data set with original processing parameters. The larger stream file combines the results of indexing with four different sets of integration radii, as described in Section 5.

	Small stream	Larger stream
Indexed patterns	66 036	247 554
Frames	125 458	479 051
File size	2.7 GB	9.7 GB
*cell_explorer*	0:20	1:16
Detector shift	0:29	1:41
*cxiview*	1:26	5:08
*DatView* preprocessing	0:44	2:42
.dat file size	61 MB	237 MB
*DatView*	0:13	0:33
